# Association of prebiotic fiber intake with colorectal cancer risk: the *PrebiotiCa* study

**DOI:** 10.1007/s00394-022-02984-y

**Published:** 2022-09-11

**Authors:** Federica Turati, Federica Concina, Marta Rossi, Federica Fiori, Maria Parpinel, Martina Taborelli, Attilio Giacosa, Anna Crispo, Eleonora Pagan, Valentina Rosato, Eva Negri, Carlo La Vecchia

**Affiliations:** 1 Unit of Medical Statistics and Biometry, Fondazione IRCCS Istituto Nazionale dei Tumori di Milano, Milan, Italy; 2 Present Address: Department of Clinical Sciences and Community Health, Università degli Studi di Milano, Milan, Italy; 3 Clinical Epidemiology and Public Health Research Unit, Institute for Maternal and Child Health-IRCCS “Burlo Garofolo”, Trieste, Italy; 4 Department of Medicine, University of Udine, Udine, Italy; 5 Unit of Cancer Epidemiology, CRO Aviano National Cancer Institute, IRCCS, Aviano, Italy; 6 Department of Gastroenterology and Clinical Nutrition, Policlinico di Monza, Monza, Italy; 7 Epidemiology Unit, Istituto Tumori “Fondazione Pascale IRCCS”, Naples, Italy; 8 Department of Statistics and Quantitative Methods, University of Milan-Bicocca, Milan, Italy; 9 Department of Medical and Surgical Sciences, University of Bologna, Bologna, Italy

**Keywords:** Colorectal cancer, Prebiotics, Fiber, Diet, Prevention

## Abstract

**Purpose:**

To evaluate the association between the intake of specific fibers with prebiotic activity, namely inulin-type fructans (ITFs), fructooligosaccharides (FOSs) and galactooligosaccharides (GOSs), and colorectal cancer risk.

**Methods:**

Within the *PrebiotiCa* study, we used data from a multicentric case–control study conducted in Italy and including 1953 incident, histologically confirmed, colorectal cancer patients and 4154 hospital controls. The amount of six prebiotic molecules [ITFs, nystose (FOS), kestose (FOS), 1F-β-fructofuranosylnystose (FOS), raffinose (GOS) and stachyose (GOS)] in a variety of foods was quantified via laboratory analyses. Subjects’ prebiotic fiber intake was estimated by multiplying food frequency questionnaire intake by the prebiotic content of each food item. The odds ratios (OR) of colorectal cancer for quintiles of intakes were derived from logistic regression models including terms for major confounders and total energy intake.

**Results:**

GOSs intake was inversely associated with colorectal cancer risk. The OR for the highest versus the lowest quintile of intake were 0.73 (95% confidence interval, CI 0.58–0.92) for raffinose and 0.64 (95% CI 0.53–0.77) for stachyose, with significant inverse trends across quintiles. No association was found with total ITFs and FOSs. The association with stachyose was stronger for colon (continuous OR = 0.74, 95% CI 0.66–0.83) than rectal cancer (OR = 0.89, 95% CI 0.79–1.02).

**Conclusion:**

Colorectal cancer risk was inversely associated with the intake of dietary GOSs, but not ITFs and FOSs.

**Supplementary Information:**

The online version contains supplementary material available at 10.1007/s00394-022-02984-y.

## Introduction

Colorectal cancer is the third most frequent incident cancer and the second cause of cancer death worldwide [1]. Besides well-described inherited genetic predisposition syndromes (e.g., familial adenomatous polyposis and Lynch syndrome), which, however, are responsible for a minority of cases [2], colorectal cancer is associated with several modifiable risk factors [3], including excess adiposity [4], physical inactivity [5] and sedentary lifestyle [6], and cigarette smoking [7], with, therefore, potential for primary prevention [8]. Diet also plays an important role in the etiology of this neoplasm. According to the World Cancer Research Fund (WCRF) and the American Institute for Cancer Research (AICR), wholegrains, food rich in dietary fiber and dairy products decrease the risk of colorectal cancer, while red and processed meat and high amounts of alcohol increase the risk [9].

The large intestine is colonized by a diverse community of microorganisms which constitutes the gut microbiota. The physiological gut microbiota has numerous functions, including gastrointestinal immune stimulation, protection against pathogens, production of essential nutrients such as vitamins, management of bioactive foods and chemical components, and modulation of gastrointestinal epithelial cell proliferation and differentiation [10]. The shift to a dysbiotic microbiota condition has been associated with the development of selected intestinal (e.g., inflammatory bowel disease) and extra-intestinal conditions (e.g., neurologic, respiratory, metabolic, hepatic, and cardiovascular diseases) [11], as well as colorectal cancer [12, 13].

Diet is one of the main drivers in shaping the gut microbiota [14, 15], potentially contributing to disease susceptibility, with habitual diets appearing to have more durable impact on the gut microbiota than short-term transient dietary strategies [16].

While it is well recognized that dietary fiber as a whole has a favorable role on gastrointestinal health, specific fiber types such as inulin-type fructans (ITFs), fructooligosaccharides (FOSs) (e.g., nystose and kestose) and galactooligosaccharides (GOSs) (e.g., raffinose and stachyose) are considered prebiotics, defined as “substrates selectively used by host microorganisms conferring health benefits” [17, 18]. These compounds bypass digestion in the small intestine and are available for bacterial fermentation in the colon; as such, they have the potential to modify the composition and metabolic activity of the gut microbiota [14, 19]. Prebiotics stimulate the growth of presumably beneficial colonic bacteria, mostly, but not exclusively, *Bifidobacterium* and *Lactobacillus* species [17, 18]. Along this line, in a recent systematic review and meta-analysis, dietary interventions involving prebiotic fibers have been associated with high abundance of *Bifidobacteria* and *Lactobacilli* [20]. Bacterial fermentation of fiber in the colonic lumen produces short chain fatty acids (SCFAs) [21], which are crucial for intestinal health [22]. In particular, the SCFA butyrate has anticarcinogenic and anti-inflammatory properties and favorable effects on colorectal carcinogenesis in animal models [23]. Fiber may also protect against colorectal cancer by increasing stool bulk, thus reducing the transit time through the bowel and the contact of carcinogens in the feces with the colonic mucosa, and by binding bile acids that produce carcinogenic metabolites [24–27].

Prebiotic fibers occur naturally in certain foods, including selected legumes, grains, fruits and vegetables, and fiber-rich foods, such as whole grains, have been reported to have a prebiotic-like effect on the gut microbiota [28]. However, still limited published data exist on the content of prebiotic fibers in foods, which derive exclusively from studies conducted outside Europe using heterogeneous methodologies [29–34].

While several investigations indicate a favorable role of high-fiber diets on colorectal cancer risk [35], whether, and which, fiber fractions with prebiotic activity contribute to this health benefit is yet to be clarified.

Within the *PrebiotiCa* (The role of prebiotics in the prevention of cancer, an integrated network of Italian case–control studies) study, we quantified in laboratory analyses selected prebiotic fibers (i.e., ITFs, nystose, kestose, 1F-β-fructofuranosylnystose, raffinose and stachyose) in a wide range of foods [36] and applied such estimates to dietary information of subjects participating in an Italian multicentric case–control study to derive subjects’ prebiotic intake. The aim of this analysis was to assess whether a diet rich in fibers with prebiotic activity may reduce colorectal cancer risk.

## Methods

### Study population

We derived data from an Italian case–control study on colorectal cancer conducted in six Italian areas (i.e., Milan, Genoa, Pordenone/Gorizia, Forlì, Latina, and Naples) in the period 1992–1996 [37]. The study included 1953 histologically confirmed colorectal cancer cases diagnosed no longer than 1 year before the interview (1225 colon cancers and 728 rectal cancers, 1125 men and 828 women, median age 62, range 19–74 years), with no previous cancer diagnosis, and 4154 controls (median age 58, range 19–74 years). Controls were subjects with no history of cancer admitted to the same hospitals as cases for acute, non-neoplastic conditions unrelated to tobacco, alcohol, hormonal or digestive tract diseases and to long-term modifications of diet; 21% were admitted for traumas, 26% for other orthopedic disorders, 24% for surgical conditions, 18% for eye diseases, and 11% for other illnesses.

The study protocol was approved by the local ethical committees, and all participants signed an informed consent. Less than 5% of either cases or controls approached refused to participate.

### Data collection

Trained interviewers administered face-to-face a structured questionnaire to cases and controls collecting data on socio-demographic characteristics, anthropometric measures, physical activity, lifetime smoking and alcohol-drinking habits, personal medical history, and family history of cancer.

An interviewer-administered food frequency questionnaire (FFQ) was used to assess study participants’ usual diet during the 2 years prior to cancer diagnosis (for cases) or hospital admission (for controls). The FFQ included the average weekly consumption of 78 foods, food groups or complex recipes; intakes lower than once a week, but at least once a month, were coded as 0.5 per week. Additional questions aimed at assessing fat intake and general dietary habits. From these data, the intakes of selected nutrients, food components, and total energy were estimated using an Italian food composition database [38] using a standardized methodology [39]; data from laboratory analysis, coducted specifically for the present project, were used for estimating subjects’ prebiotic intake. The FFQ was tested for reproducibility [37, 40] and validity [38]. As for reproducibility, correlation coefficients between intakes estimated by two FFQ were 0.67 for fiber, and between 0.6 and 0.7 for most of the FFQ items in the “bread, cereals and first courses” category, between 0.5 and 0.6 for most of the vegetables, root vegetables, tubers roots and legumes, and around 0.6–0.7 for various fruits. In the validation study, the correlation coefficient between the intakes estimated from the FFQ and from two 7-day diaries was 0.58 of fibers, around 0.60–0.65 for energy, available carbohydrates, sugar and starch, around 0.50 for total, animal and vegetable proteins, animal fats and saturated fatty acids.

### Quantification of prebiotic fibers in foods

The methodology used for the quantification of prebiotic fibers was described in details [36]. In brief, FOSs (i.e., nystose [glucose–3*fructose], kestose [glucose–2*fructose] and 1F-β-fructofuranosylnystose [glucose–4*fructose]) and GOSs (raffinose [galactose–glucose–fructose] and stachyose [2*galactose–glucose–fructose]) were determined in 78 food sources; ITFs in 7. Food sampling and analysis were conducted at the laboratory of Neotron SpA in Modena, which has a certified laboratory for food analysis. The food products investigated included 15 types of fruits, 32 varieties of vegetables, root vegetables and tubers, 9 types of dried or fresh legumes, and 22 cereals and cereals-based products (both wholegrain and refined products). Most of these were included in the FFQ used in the present case–control study (as a specific FFQ item, as a food of an item including mixed foods, or as a food ingredient of an item consisting of complex recipes). The 78 samples (unique samples) analyzed in this study were collected from supermarkets located in Modena from 17 May to 24 June 2021.

ITFs were determined using an internal analytical method based on AOAC 997.08 procedure. Freeze-dried samples were extracted in hot water (*T* equal to 85 °C) with mild agitation and the pH checked immediately (pH equal to 6.5–8.0, at 85 °C) (extract *A*_0_). A portion of extracted *A*_0_ was first hydrolyzed with a sufficient amount of amyloglucosidase solution, taking into account amount of starch and maltodextrins present (extract *A*_1_), and second hydrolyzed with a sufficient amount of inulinase solution, taking into account amount of fructans present, and enzyme concentration (Fructozyme) (extract *A*_2_). Extract *A*_0_, *A*_1_ and *A*_2_ were injected into a high-performance anion-exchange chromatography coupled to pulsed amperometric detection (HPAE-PAD), previous addition of 2.0 g of glucoheptose internal standard solution in order to determine the following sugars: glucose, fructose, sucrose, maltitol and galactose, and calculate ITFs content using a specific formula. ITFs were determined in fresh onion, garlic, banana, leek, Jerusalem artichoke, artichoke and shallot. The analysis was performed based on a limit of detection (LOD) of the methodology equal to less than 0.005. ITFs content ranged from 25.1 g/100 g in garlic to 1 g/100 g in onion and leek.

FOSs and GOSs in fresh samples were determined according to Manali Aggrawal and Jeff Rohrer method (Thermo Scientific, Application Note 1149: Profiling Fructosyloligosaccharides (FOS)-containing samples by HPAE-PAD. Sunnyvale, CA, 2015). One gram of homogenized sample was extracted with 200 mL of sodium hydroxide 0.0025 M and then analyzed using HPAE-PAD method. The LOD was between < 0.002 and < 0.010. The following molecules were quantified: raffinose (GOS), stachyose (GOS), nystose (FOS), kestose (FOS) and 1F-fructofuranosylnystose (FOS). Total FOSs was calculated as the sum of nystose, kestose and 1F-β-fructofuranosylnystose.

The food with the highest content of FOSs was Jerusalem artichoke (4.45 g/100 g), with other foods containing less than 1 g/100 g; FOSs were represented principally as kestose. Pulses, excluding green beans, had the highest content of GOSs, with a mean content of 1.17 ± 0.87 g/100 g. In particular, raffinose was particularly abundant in dried peas (0.498 g/100 g) and chickpeas (0.463 g/100 g) and stachyose in dried beans (1.905 g/100 g) and peas (1.814 g/100 g) [36].

### Data analysis

We derived the odds ratios (OR) of colorectal cancer with the corresponding 95% confidence intervals (CI) according to quintiles (computed among controls) of fiber prebiotic intake by multiple logistic regression models, adjusted for age, sex, study center, years of education (< 7, 7–11, > 11), body mass index (BMI, in quintiles), occupational physical activity (low, medium, high), smoking habits (never, former, current of < 15, current of ≥ 15 cigarettes/day), alcohol intake (in quartiles), age at menopause and use of hormone replacement therapy, diabetes, aspirin use, family history of colorectal cancer, and total energy intake (in quintiles). In a sensitivity analysis, we included in the model additional terms for total fiber intake when the association estimated from the main model was significant. Tests for trends across quintiles were performed by including the examined variable as ordinal. In addition, prebiotics were entered into the models as continuous variables, with a measurement unit equal to the difference between the upper cutpoints of the 4th (i.e., the 80th percentile) and the 1st quintile (i.e., the 20th percentile). We also assessed the associations using “calorie-adjusted” prebiotic intakes, calculated according to the residual method [41]. Stratification for age, sex and BMI and separate analysis by colorectal cancer subsites were performed for prebiotic fibers showing significant association with colorectal cancer. Heterogeneity across strata was evaluated by testing the significance of the product term between the exposure variable in continuous and the dichotomous stratification factor. Heterogeneity across subsites (colon and rectum) was tested using the Wald test.

All the analyses were performed using the SAS software, version 9.4 (SAS Institute, Inc., Cary, NC, USA).

## Results

Table 1 gives the distribution of colon and rectal cancer cases and controls by sex, age, and other selected factors. Both colon and rectal cancer cases reported more frequently family history of intestinal cancer than controls. Colon, but not rectal, cancer cases were more educated than controls and reported more frequently a low level of physical activity.

**Table 1:** Distribution of 1225 cases of colon cancer, 728 cases of rectal cancer and 4154 controls, according to sex, age and other factors. Italy, 1992–1996

Characteristics	Cancers cases			*p* value^b^ colon cancer versus controls	*p* value^b^ rectal cancer versus controls
	Colon	Rectum	Controls		
	Number (%)	number (%)	number (%)		
Sex					
Men	688 (56.2)	437 (60.0)	2073 (49.9)		
Women	537 (43.8)	291 (40.0)	2081 (50.1)	< 0.001	< 0.001
Age (years)					
< 40	55 (4.5)	26 (3.6)	347 (8.4)		
40–49	114 (9.3)	67 (9.2)	732 (17.6)		
50–59	321 (26.2)	197 (27.1)	1244 (30.0)		
60–69	518 (42.3)	306 (42.0)	1356 (32.6)		
≥ 70	217 (17.7)	132 (18.1)	475 (11.4)	< 0.001	< 0.001
Education^a^ (years)					
< 7	621 (50.9)	422 (58.2)	2276 (55.2)		
7–11	331 (27.2)	181 (25.0)	1156 (28.0)		
≥ 12	267 (21.9)	122 (16.8)	693 (16.8)	< 0.001	0.211
Occupational physical activity^a^					
Low	468 (38.2)	236 (32.4)	1351 (32.5)		
Medium	433 (35.4)	255 (35.0)	1578 (37.6)		
High	324 (26.5)	237 (32.6)	1224 (29.5)	0.001	0.181
Family history of intestinal cancer					
No	1091 (89.1)	675 (92.7)	4008 (96.5)		
Yes	134 (10.9)	53 (7.3)	146 (3.5)	< 0.001	< 0.001

^a^The sum does not add up to the total because of some missing values

^b^From Chi-square test

Among controls, median daily intakes (mg) were 798 for ITFs, 167 for kestose, 16 for nystose, 2 for 1F-β-fructofuranosylnystose, 94 for raffinose, and 180 for stachyose.

The intake of prebiotic fibers was positively correlated with total fiber intake. Pearson correlation coefficients were 0.31 with total ITFs, 0.49 with kestose, 0.36 with nystose, 0.70 with 1F-β-fructofuranosylnystose, 0.70 with total FOSs, 0.72 with raffinose and 0.45 with stachyose. Kestose was the largest contributor to total FOSs intake (89.7%); nystose (7.9%) and 1F-β-fructofuranosylnystose (2.4%) accounted for a small fraction of total FOSs intake.

Supplementary Table 1 shows the distribution of potential confounders according to quintiles of prebiotic intake among controls. Controls with higher prebiotic intake were less frequently women, had higher total energy intake, reported less frequently history of diabetes, and, with the exception of stachyose, tended to be younger. Moreover, subjects with higher intake of ITFs tended to have a lower level of physical activity and to be more frequently current smokers; those with higher intake of raffinose and total FOSs tended to be more frequently alcohol drinkers. Women with higher ITFs, raffinose or total FOSs intakes were less frequently in menopause.

Table 2 provides the OR of colorectal cancer, and the corresponding 95% CI, according to prebiotic intake. Inverse associations were observed with the intakes of GOSs. The continuous OR were 0.85 (95% CI 0.76–0.96) for raffinose and 0.81 (95% CI 0.74–0.89) for stachyose. The OR for the highest versus the lowest quintile were 0.73 (95% CI 0.58–0.92) for raffinose and 0.64 (95% CI 0.53–0.77) for stachyose, with significant trends of decreasing risk across quintiles. After further adjustment for total fiber intake was performed, the association with raffinose intake attenuated (OR for Q_5_ vs. Q_1_: 0.80, 95% CI 0.62–1.04, *p* for trend across quintiles: 0.067) while that with stachyose intake remained virtually unchanged (OR for Q_5_ vs. Q_1_: 0.66, 95% CI, 0.54–0.80, *p* for trend across quintiles: < 0.001). Similar OR were obtained when using “calorie-adjusted” intakes.

**Table 2:** Odds ratios (OR) of colorectal cancer and corresponding 95% confidence intervals (CI) according to the intake of selected prebiotic fibers. Italy, 1992–1996

	Quintiles^a^					*P*_trend_	Continuous^b^
	Q1	Q2	Q3	Q4	Q5		
Inulin-type fructans (mg)							
Upper cutpoint	377	642	978	1705	–		
Cases (%)	393 (20.1)	402 (20.6)	389 (19.9)	349 (17.9)	420 (21.5)		
OR^c^ (95% CI)	1^d^	0.92 (0.77–1.09)	0.87 (0.72–1.04)	0.83 (0.69–1.00)	0.96 (0.80–1.16)	0.370	0.99 (0.94–1.04)
Kestose (FOS) (mg)							
Upper cutpoint	120	152	183	230	–		
Cases (%)	362 (18.5)	380 (19.5)	415 (21.2)	389 (19.9)	407 (20.8)		
OR^c^ (95% CI)	1^d^	0.99 (0.82–1.19)	1.05 (0.86–1.28)	0.95 (0.77–1.16)	0.97 (0.78–1.21)	0.484	0.97 (0.88–1.07)
Nystose (FOS) (mg)							
Upper cutpoint	11	14	17	21	–		
Cases (%)	325 (16.6)	396 (20.3)	401 (20.5)	399 (20.4)	432 (22.1)		
OR^c^ (95% CI)	1^d^	1.18 (0.98–1.42)	1.18 (0.97–1.43)	1.16 (0.94–1.41)	1.22 (0.98–1.51)	0.060	1.07 (0.96–1.20)
1F-β-Fructofuranosylnystose (FOS) (mg)							
Upper cutpoint	0.8	1.9	3.1	8.0	–		
Cases (%)	414 (21.2)	351 (18.0)	358 (18.3)	400 (20.5)	430 (22.0)		
OR^c^ (95% CI)	1^d^	0.84 (0.70–1.00)	0.83 (0.70–1.00)	0.89 (0.74–1.07)	0.99 (0.83–1.19)	0.984	0.97 (0.92–1.03)
Total FOSs (mg)							
Upper cutpoint	134	170	205	257	–		
Cases (%)	344 (17.6)	390 (20.0)	421 (21.6)	392 (20.1)	406 (20.8)		
OR^c^ (95% CI)	1^d^	1.08 (0.90–1.31)	1.14 (0.94–1.39)	1.03 (0.83–1.27)	1.03 (0.83–1.29)	0.856	0.97 (0.88–1.08)
Raffinose (GOS) (mg)							
Upper cutpoint	68	85	102	125	–		
Cases (%)	377 (19.3)	404 (20.7)	425 (21.8)	379 (19.4)	368 (18.8)		
OR^c^ (95% CI)	1^d^	0.99 (0.82–1.19)	0.98 (0.81–1.19)	0.83 (0.68–1.03)	0.73 (0.58–0.92)	0.002	0.85 (0.76–0.96)
OR^e^ (95% CI)	1^d^	1.06 (0.88–1.28)	1.09 (0.88–1.34)	0.92 (0.73–1.16)	0.80 (0.62–1.04)	0.067	0.90 (0.79–1.02)
Stachyose (GOS) (mg)							
Upper cutpoint	93	163	224	341	–		
Cases (%)	444 (22.7)	425 (21.8)	375 (19.2)	347 (17.8)	362 (18.5)		
OR^c^ (95% CI)	1^d^	0.90 (0.76–1.06)	0.77 (0.65–0.92)	0.65 (0.55–0.78)	0.64 (0.53–0.77)	< 0.001	0.81 (0.74–0.89)
OR^e^ (95% CI)	1^d^	0.90 (0.76–1.08)	0.79 (0.66–0.94)	0.67 (0.56–0.81)	0.66 (0.54–0.80)	< 0.001	0.83 (0.75–0.92)

*FOS* fructooligosaccharide, *GOS* galactooligosaccharides

^a^Derived among controls

^b^OR for an increase of intake equal to the difference between the upper cutpoints of the 4th and the 1st quintiles

^c^Adjusted for age, sex, study center, education, body mass index, physical activity, smoking habits, alcohol intake, age at menopause and use of hormone replacement therapy, diabetes, aspirin use, family history of intestinal cancer, and total energy intake

^d^Reference category

^e^Further adjusted for total fiber intake

Total ITFs and compounds of the FOSs family were not associated with colorectal cancer.

**Fig. 1:**
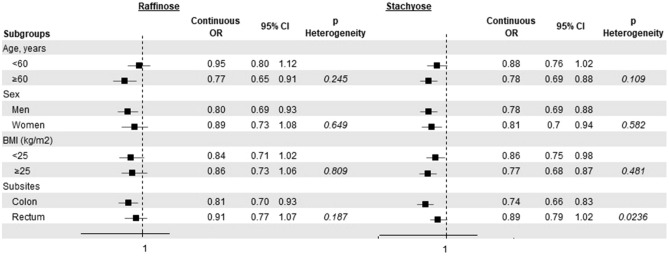
Odds ratios (OR)^a^ of colorectal cancer and corresponding 95% confidence intervals (CI) according to raffinose and stachyose intake in strata of age, sex, body mass index and colorectal cancer subsites. Italy, 1992–1996. ^a^OR for an increase of intake equal to the difference between the upper cutpoints of the 4th (i.e., the 80th percentile) and the 1st quintiles (i.e., the 20th percentile), adjusted for age, sex, study center, education, body mass index (BMI), physical activity, smoking habits, alcohol intake, age at menopause and use of hormone replacement therapy, diabetes, regular aspirin use, family history of intestinal cancer, and total energy intake, unless the variable was the stratification factor

In subgroup analyses on raffinose and stachyose intakes (Fig. 1), slight variations in the strength of the (inverse) associations were observed, without significant heterogeneity between strata. When colorectal cancer subsites where analyzed separately, the association with stachyose intake was stronger for colon (continuous OR = 0.74, 95% CI 0.66–0.83) than rectal cancer (continuous OR = 0.89, 95% CI 0.79–1.02).

## Discussion

Within the *PrebiotiCa* study, we determined the intake of six fiber fractions with prebiotic activity in subjects participating in an Italian case–control study by means of novel analytical food composition data and evaluated whether high dietary intakes of such prebiotic fibers influence the risk of colorectal cancer. Our results suggest an inverse association with dietary GOSs intake (particularly for colon cancer), but no association with total ITFs and prebiotic fibers of the FOSs family. Although the association with raffinose intake was attenuated after adjustment for total fiber, the association with stachyose intake remained virtually unchanged, suggesting that part of the protection afforded by fiber consumption on colorectal cancer may be through prebiotic effects. The observed variations in the strength of the GOSs-colorectal cancer association among subgroups of the population may be attributed to chance, in the absence of significant heterogeneity across strata.

In animal studies, ITFs showed anticarcinogenic properties [42]. Besides the extremely different setting, which limits any direct comparison with animal research, our study relies with low intakes of ITFs. Indeed, these compounds were quantified in a limited number of foods, seldom consumed and in low amounts. Whether a favorable effect of higher intakes of ITFs exists cannot, therefore, be excluded.

While a wealth of data is available on the role of fiber on colorectal cancer risk [20] and on its potential to modify the gut microbiota composition [15, 16], epidemiological studies did not evaluate the association with naturally occurring dietary prebiotics. Prebiotic fiber supplement use was not associated with a reduced risk of colorectal cancer in a cohort study of post-menopausal women [43]. Nutritional guidelines for cancer prevention by the WCRF and the AICR are not encouraging the use of dietary supplementation, but instead recommend that nutritional needs be met primarily through food consumption [44].

In laboratory analyses, GOSs were abundant in legumes. In particular, raffinose was abundant in dried peas and chickpeas (~ 0.46–0.5 g/100 g); stachyose in dried beans, peas, and chickpeas (~ 1.6–1.9 g/100 g) followed by lentils, fresh peas and fresh chickpeas (~ 0.27–0.41 g/100 g). While stachyose was detected in significant amounts in legumes only, other foods containing raffinose included whole meal flour (0.3 g/100 g) and selected wholegrain-based products (e.g., wholemeal biscuits, wholemeal pasta) (~ 0.2/100 g), as well as barley (0.22 g/100 g); raffinose was detected also in white wheat flour and wheat products, but in lower amounts (0.04 g/100 g). As for dietary FOSs, kestose was abundant in shallot, garlic, whole wheat pasta, wholemeal biscuits, banana and barley (range, ~ 0.54–0.15 g/100 g); low or undetectable amounts of kestose were found in legumes. Nystose and 1F-β-fructofuranosylnystose were detected in small concentrations in a few foods (e.g., shallot, garlic and barley) [36].

Accounting for amount consumed, the largest contributors of raffinose and stachyose in our population were cereal-based products and legumes (for stachyose, mainly legumes), followed by vegetables and fruit, which provided limited contributions; bananas were the most important source of ITFs, accounting alone for almost 60% of the intake; as for FOSs, kestose came mainly from cereals and fruit in a similar proportion. The few food products containing nystose and 1F-β-fructofuranosylnystose were consumed in small amounts and low daily intakes of these prebiotic fibers were estimated in our database, with limited variation across subjects.

Supporting our results, the intakes of wholegrain products and fiber from grains have been favorably associated with the risk of colorectal cancer in various studies [45–47]. An association with grain fiber intake was observed even in studies in which no association with total fiber was detected [48–50]. The effect of legume intakes on colorectal cancer intake was less clear. According to a meta-analysis published in 2015 and based on 14 cohort studies, higher legumes consumption was associated with a significant 10% reduced risk of colorectal cancer [51]. A 2018 meta-analysis based on a partially overlapping set of studies of the 2015 meta-analysis (for a total 14 cohort studies) found, however, no association with legumes for high versus low intake and no dose–response relation [47]. The intake of fiber from legumes was associated with a non-significant 16% decreased risk of colorectal cancer in a meta-analysis of 6 studies published in 2019 [45]. The intake of fruit and fruit fiber is not appreciably associated with colorectal cancer risk [35, 45, 50, 52].

Some limitations of the study are inherent in the study design. With reference to possible selection bias, our study was not population-based, but cases were identified in the major teaching and general hospitals of the area under surveillance, and the participation was almost complete. As for controls, these were from comparable catchment areas as cases and their participation rate was high and similar to that of cases (> 95%); further, we excluded patients admitted for chronic conditions or for diseases related to diet modifications or known risk factors for colorectal cancer. The similar interview setting of cases and controls provides reassurance against potential information bias. Recall bias and measurement error in dietary assessment using an FFQ is difficult to avoid in a case–control study. However, the FFQ gave satisfactory results when tested for reproducibility and validity of fiber intake and other food components [38, 40]. With regard to confounding, we were able to consider a number of possible confounding factors in the analysis. The intake of selected prebiotics, namely ITFs and raffinose, appeared associated with some risk factors for colorectal cancer (e.g., low physical activity, current smoking and alcohol intake); such potential confounders were taken into account in multiple adjusted models. Any residual confounding may have led, if anything, to underestimation of the association with raffinose intake or masking an association with ITFs. Allowance for total fiber intake may be considered an over-adjustment, and is provided as a sensitivity analysis. OR without allowance for total fiber give more valid estimates. Another limitation relates to the application of results from food content analyses conducted in 2021 to dietary intakes collected in the 1990s’. However, no prior data on the prebiotic content of Italian foods were available and those from other countries were limited and scattered across studies using different methodologies. Linking dietary data to food composition data collected at different time points, when contemporary data are not available, is a common approach in nutritional studies [53].

Accurate estimation of dietary prebiotic intake may be challenging due to the lack of published food composition data and heterogeneity in methodologies. Furthermore, the definition of ITFs is not universally agreed. For the purpose of our study, we quantified through laboratory analysis the amount of ITFs, nystose, kestose, 1F-β-fructofuranosylnystose, raffinose and stachyose in individual foods (e.g., flour, specific legumes, fruits and vegetables) as well as in recipes (e.g., bread, pasta, biscuits) and applied such determinations to the self-reported weekly frequency of consumption of foods, groups of foods, or recipes obtained from the FFQ. When a single item included different foods or recipes, these were assigned a relative proportion according to nationally representative data. The FFQ used for the present study was not specifically designed to measure the intake of prebiotic fibers and, although the main sources of prebiotics were addressed, it did not include items on certain foods rich in prebiotic fibers such as rye products, spelt, Jerusalem artichoke, breakfast cereal products, oats and soya beans [29–34], which, however, are infrequently consumed in Italy. In addition, the FFQ distinguished between wholegrain and non-wholegrain only for bread. In any case, possible misclassification of prebiotic intake should not be unbalanced between cases and controls.

Total ITFs was only quantified in 6 foods assessed in our FFQ, with garlic having by far the highest content. The FFQ collected information on usual garlic consumption in three categories: nonuse or low use, intermediate use, and high use. In our main analysis, we estimated subject’s garlic intake based on a standard amount of garlic in each recipe. When, in an additional sensitivity analysis, we weighted subject’s garlic intake based on the reported qualitative indicator of consumption (i.e., multiplying the intake by 0.2 for nonuse or low use and by 1.8 for high use), we obtained a slightly lower mean total ITFs intake, and similar OR for the association between ITFs intake and colorectal cancer (data not shown).

In conclusion, the present study suggests an inverse association between selected prebiotic fibers, i.e., those of the GOSs family, and colorectal cancer risk.

## Supplementary Information

Below is the link to the electronic supplementary material.

Supplementary file1 (PDF 335 KB)

## References

[1] Sung H, Ferlay J, Siegel RL, Laversanne M, Soerjomataram I, Jemal A, Bray F (2021). Global cancer statistics 2020: GLOBOCAN estimates of incidence and mortality worldwide for 36 cancers in 185 countries. CA Cancer J Clin.

[2] Foulkes WD (2008). Inherited susceptibility to common cancers. N Engl J Med.

[3] Keum N, Giovannucci E (2019). Global burden of colorectal cancer: emerging trends, risk factors and prevention strategies. Nat Rev Gastroenterol Hepatol.

[4] Freisling H, Arnold M, Soerjomataram I, O'Doherty MG, Ordonez-Mena JM, Bamia C, Kampman E, Leitzmann M, Romieu I, Kee F, Tsilidis K, Tjonneland A, Trichopoulou A, Boffetta P, Benetou V, Bueno-de-Mesquita HBA, Huerta JM, Brenner H, Wilsgaard T, Jenab M (2017). Comparison of general obesity and measures of body fat distribution in older adults in relation to cancer risk: meta-analysis of individual participant data of seven prospective cohorts in Europe. Br J Cancer.

[5] Boyle T, Keegel T, Bull F, Heyworth J, Fritschi L (2012). Physical activity and risks of proximal and distal colon cancers: a systematic review and meta-analysis. J Natl Cancer Inst.

[6] Schmid D, Leitzmann MF (2014). Television viewing and time spent sedentary in relation to cancer risk: a meta-analysis. J Natl Cancer Inst.

[7] Botteri E, Borroni E, Sloan EK, Bagnardi V, Bosetti C, Peveri G, Santucci C, Specchia C, van den Brandt P, Gallus S, Lugo A (2020). Smoking and colorectal cancer risk, overall and by molecular subtypes: a meta-analysis. Am J Gastroenterol.

[8] Brenner H, Chen C (2018). The colorectal cancer epidemic: challenges and opportunities for primary, secondary and tertiary prevention. Br J Cancer.

[9] World Cancer Research Fund/American Institute for Cancer Research. Continuous Update Project Expert Report 2018. Diet, nutrition, physical activity and colorectal cancer. Available at dietandcancerreport.org

[10] Lynch SV, Pedersen O (2016). The human intestinal microbiome in health and disease. N Engl J Med.

[11] Hand TW, Vujkovic-Cvijin I, Ridaura VK, Belkaid Y (2016). Linking the microbiota, chronic disease, and the immune system. Trends Endocrinol Metab.

[12] Wong SH, Yu J (2019). Gut microbiota in colorectal cancer: mechanisms of action and clinical applications. Nat Rev Gastroenterol Hepatol.

[13] Tilg H, Adolph TE, Gerner RR, Moschen AR (2018). The intestinal microbiota in colorectal cancer. Cancer Cell.

[14] David LA, Maurice CF, Carmody RN, Gootenberg DB, Button JE, Wolfe BE, Ling AV, Devlin AS, Varma Y, Fischbach MA, Biddinger SB, Dutton RJ, Turnbaugh PJ (2014). Diet rapidly and reproducibly alters the human gut microbiome. Nature.

[15] Kolodziejczyk AA, Zheng D, Elinav E (2019). Diet-microbiota interactions and personalized nutrition. Nat Rev Microbiol.

[16] Leeming ER, Johnson AJ, Spector TD, Le Roy CI (2019). Effect of diet on the gut microbiota: rethinking intervention duration. Nutrients.

[17] Gibson GR, Hutkins R, Sanders ME, Prescott SL, Reimer RA, Salminen SJ, Scott K, Stanton C, Swanson KS, Cani PD, Verbeke K, Reid G (2017). Expert consensus document: The International Scientific Association for Probiotics and Prebiotics (ISAPP) consensus statement on the definition and scope of prebiotics. Nat Rev Gastroenterol Hepatol.

[18] Verspreet J, Damen B, Broekaert WF, Verbeke K, Delcour JA, Courtin CM (2016). A critical look at prebiotics within the dietary fiber concept. Annu Rev Food Sci Technol.

[19] Backhed F, Ley RE, Sonnenburg JL, Peterson DA, Gordon JI (2005). Host-bacterial mutualism in the human intestine. Science.

[20] Reynolds A, Mann J, Cummings J, Winter N, Mete E, Te Morenga L (2019). Carbohydrate quality and human health: a series of systematic reviews and meta-analyses. Lancet.

[21] Holscher HD (2017). Dietary fiber and prebiotics and the gastrointestinal microbiota. Gut Microbes.

[22] Marchesi JR, Adams DH, Fava F, Hermes GD, Hirschfield GM, Hold G, Quraishi MN, Kinross J, Smidt H, Tuohy KM, Thomas LV, Zoetendal EG, Hart A (2016). The gut microbiota and host health: a new clinical frontier. Gut.

[23] Goncalves P, Martel F (2013). Butyrate and colorectal cancer: the role of butyrate transport. Curr Drug Metab.

[24] Lupton JR, Turner ND (1999). Potential protective mechanisms of wheat bran fiber. Am J Med.

[25] Cummings JH, Bingham SA, Heaton KW, Eastwood MA (1992). Fecal weight, colon cancer risk, and dietary intake of nonstarch polysaccharides (dietary fiber). Gastroenterology.

[26] Harris PJ, Ferguson LR (1993). Dietary fibre: its composition and role in protection against colorectal cancer. Mutat Res.

[27] Kritchevsky D (1995). Epidemiology of fibre, resistant starch and colorectal cancer. Eur J Cancer Prev.

[28] Costabile A, Klinder A, Fava F, Napolitano A, Fogliano V, Leonard C, Gibson GR, Tuohy KM (2008). Whole-grain wheat breakfast cereal has a prebiotic effect on the human gut microbiota: a double-blind, placebo-controlled, crossover study. Br J Nutr.

[29] Muir JG, Shepherd SJ, Rosella O, Rose R, Barrett JS, Gibson PR (2007). Fructan and free fructose content of common Australian vegetables and fruit. J Agric Food Chem.

[30] Biesiekierski JR, Rosella O, Rose R, Liels K, Barrett JS, Shepherd SJ, Gibson PR, Muir JG (2011). Quantification of fructans, galacto-oligosacharides and other short-chain carbohydrates in processed grains and cereals. J Hum Nutr Diet.

[31] Moshfegh AJ, Friday JE, Goldman JP, Ahuja JK (1999). Presence of inulin and oligofructose in the diets of Americans. J Nutr.

[32] Campbell JM, Bauer LL, Fahey GC, Hogarth A, Wolf BW, Hunter DE (1997). Selected fructooligosaccharide (1-kestose, nystose, and 1F-β-fructofuranosylnystose) composition of foods and feeds. J Agric Food Chem.

[33] Hogarth AJ, Hunter DE, Jacobs WA, Garleb KA, Wolf BW (2000). Ion chromatographic determination of three fructooligosaccharide oligomers in prepared and preserved foods. J Agric Food Chem.

[34] Judprasong K, Tanjor S, Puwastien P, Sungpuag P (2011). Investigation of Thai plants for potential sources of inulin-type fructans. J Food Compos Anal.

[35] Aune D, Chan DS, Lau R, Vieira R, Greenwood DC, Kampman E, Norat T (2011). Dietary fibre, whole grains, and risk of colorectal cancer: systematic review and dose-response meta-analysis of prospective studies. BMJ.

[36] Fiori F, Concina F, Turati F, Meschiari M, Gaboardi G, Galli F, La Vecchia C, Parpinel M (2022). Quantification of naturally occurring prebiotic fiber in italian foods. J Food Compos Anal.

[37] Franceschi S, Favero A, La Vecchia C, Negri E, Conti E, Montella M, Giacosa A, Nanni O, Decarli A (1997). Food groups and risk of colorectal cancer in Italy. Int J Cancer.

[38] Decarli A, Franceschi S, Ferraroni M, Gnagnarella P, Parpinel MT, La Vecchia C, Negri E, Salvini S, Falcini F, Giacosa A (1996). Validation of a food-frequency questionnaire to assess dietary intakes in cancer studies in Italy. Results for specific nutrients. Ann Epidemiol.

[39] Westenbrink S, Oseredczuk M, Castanheira I, Roe M (2009). Food composition databases: the EuroFIR approach to develop tools to assure the quality of the data compilation process. Food Chem.

[40] Franceschi S, Negri E, Salvini S, Decarli A, Ferraroni M, Filiberti R, Giacosa A, Talamini R, Nanni O, Panarello G (1993). Reproducibility of an Italian food frequency questionnaire for cancer studies: results for specific food items. Eur J Cancer.

[41] Willett W, Stampfer MJ (1986). Total energy intake: implications for epidemiologic analyses. Am J Epidemiol.

[42] Pool-Zobel BL (2005). Inulin-type fructans and reduction in colon cancer risk: review of experimental and human data. Br J Nutr.

[43] Skiba MB, Kohler LN, Crane TE, Jacobs ET, Shadyab AH, Kato I, Snetselaar L, Qi L, Thomson CA (2019). The association between prebiotic fiber supplement use and colorectal cancer risk and mortality in the women's health initiative. Cancer Epidemiol Biomarkers Prev.

[44] World Cancer Research Fund/American Institute for Cancer Research. Continuous Update Project Expert Report 2018. Recommendations and public health policy implications. Available at dietandcancerreport.org

[45] Oh H, Kim H, Lee DH, Lee A, Giovannucci EL, Kang SS, Keum N (2019). Different dietary fibre sources and risks of colorectal cancer and adenoma: a dose-response meta-analysis of prospective studies. Br J Nutr.

[46] Larsson SC, Giovannucci E, Bergkvist L, Wolk A (2005). Whole grain consumption and risk of colorectal cancer: a population-based cohort of 60,000 women. Br J Cancer.

[47] Schwingshackl L, Schwedhelm C, Hoffmann G, Knuppel S, Laure Preterre A, Iqbal K, Bechthold A, De Henauw S, Michels N, Devleesschauwer B, Boeing H, Schlesinger S (2018). Food groups and risk of colorectal cancer. Int J Cancer.

[48] He X, Wu K, Zhang X, Nishihara R, Cao Y, Fuchs CS, Giovannucci EL, Ogino S, Chan AT, Song M (2019). Dietary intake of fiber, whole grains and risk of colorectal cancer: an updated analysis according to food sources, tumor location and molecular subtypes in two large US cohorts. Int J Cancer.

[49] Hullings AG, Sinha R, Liao LM, Freedman ND, Graubard BI, Loftfield E (2020). Whole grain and dietary fiber intake and risk of colorectal cancer in the NIH-AARP Diet and Health Study cohort. Am J Clin Nutr.

[50] Bradbury KE, Murphy N, Key TJ (2020). Diet and colorectal cancer in UK Biobank: a prospective study. Int J Epidemiol.

[51] Zhu B, Sun Y, Qi L, Zhong R, Miao X (2015). Dietary legume consumption reduces risk of colorectal cancer: evidence from a meta-analysis of cohort studies. Sci Rep.

[52] Vieira AR, Abar L, Chan DSM, Vingeliene S, Polemiti E, Stevens C, Greenwood D, Norat T (2017). Foods and beverages and colorectal cancer risk: a systematic review and meta-analysis of cohort studies, an update of the evidence of the WCRF-AICR Continuous Update Project. Ann Oncol.

[53] Willett W (2012) Nutritional epidemiology. Oxford University Press, Oxford, ISBN 9780199754038

