# Supplementary material for: Association of prebiotic fiber intake with colorectal cancer risk: the PrebiotiCa study
Source: Eur J Nutr. 2022 Sep 11;62(1):455–64. doi: 10.1007/s00394-022-02984-y (PMC9899730; doi:10.1007/s00394-022-02984-y)
Supplement: Supplementary file 1 — Supplementary file1 (PDF 335 KB) [file 394_2022_2984_MOESM1_ESM.pdf]

Supplementary Table 1. Distribution of selected factors according to quintiles of Inulin-type fructans, raffinose, stachyose and total fructo-oligosaccharides (FOS) among 4155 controls. Italy, 1992-1996.

	Quintiles of intake				
	Q1	Q2	Q3	Q4	Q5
Inulin-type fructans (ITFs)					
Women (%)	442 (53.2)	437 (52.6)	406 (48.9)	423 (51.0)	373 (44.9)
Age, years*	57.1 (10.9)	57.6 (9.9)	57.7 (10.5)	55.4 (11.5)	53.8 (12.7)
Body mass index*, kg/m^2	26.2 (4.1)	26.1 (3.9)	26.2 (4.0)	25.6 (3.9)	24.9 (3.8)
Medium/high physical activity (%)	612 (73.7)	574 (69.1)	572 (68.8)	535 (64.5)	509 (61.3)
Current smokers (%)	227 (27.3)	225 (27.1)	222 (26.7)	262 (31.6)	286 (34.4)
Alcohol drinkers (%)	636 (76.6)	638 (76.8)	658 (79.3)	648 (78.5)	650 (78.3)
Postmenopausal status (in women) (%)	318 (72.0)	330 (75.5)	286 (70.4)	275 (65.0)	224 (60.1)
Diabetes (%)	42 (5.1)	55 (6.6)	51 (6.1)	21 (2.5)	12 (1.4)
Regular aspirin use (%)	13 (1.6)	14 (1.7)	10 (1.2)	11 (1.3)	17 (2.1)
Family history of intestinal cancer (%)	25 (3.0)	29 (3.5)	33 (4.0)	24 (2.9)	35 (4.2)
Total energy intake*, kcal/day	2089 (750)	2326 (765)	2441 (763)	2509 (781)	2769 (943)
Raffinose (GOS)					
Women (%)	560 (67.5)	444 (53.4)	399 (48.0)	355 (42.7)	323 (38.9)
Age, years*	57.2 (11.2)	56.8 (11.1)	56.2 (10.8)	56.1 (11.2)	55.3 (11.9)
Body mass index*, kg/m^2	25.9 (4.3)	25.9 (4.1)	25.8 (3.9)	25.8 (3.8)	25.6 (3.7)
Medium/high physical activity (%)	524 (63.1)	590 (70.9)	564 (67.9)	573 (69.0)	551 (66.5)
Current smokers (%)	237 (28.6)	257 (30.9)	238 (28.6)	240 (28.9)	250 (30.1)
Alcohol drinkers (%)	553 (66.6)	664 (80.1)	669 (80.7)	677 (81.5)	667 (80.6)
Postmenopausal status (in women) (%)	406 (72.5)	297 (66.9)	274 (68.7)	248 (69.9)	208 (64.4)
Diabetes (%)	57 (6.9)	45 (5.4)	34 (4.1)	32 (3.9)	13 (1.6)
Regular aspirin use (%)	11 (1.3)	11 (1.3)	15 (1.8)	16 (1.9)	12 (1.5)
Family history of intestinal cancer (%)	32 (3.9)	25 (3.0)	28 (3.4)	34 (4.1)	27 (3.3)
Total energy intake*, kcal/day	1684 (522)	2131 (567)	2398 (569)	2646 (620)	3276 (876)
Stachyose (GOS)					
Women (%)	469 (56.4)	410 (49.4)	419 (50.4)	401 (48.3)	382 (46.0)
Age, years*	55.5 (12.2)	55.7 (11.4)	55.6 (11.0)	56.9 (11.1)	57.7 (10.5)
Body mass index*, kg/m^2	25.9 (4.4)	26.0 (4.0)	25.7 (3.9)	25.7 (3.8)	25.7 (3.6)
Medium/high physical activity	525 (63.2)	559 (67.4)	579 (69.7)	581 (69.9)	558 (67.2)
Current smokers (%)	253 (30.5)	247 (29.8)	253 (30.5)	229 (27.6)	240 (28.8)
Alcohol drinkers (%)	611 (73.6)	646 (78.0)	662 (79.8)	662 (79.9)	649 (78.2)
Postmenopausal status (in women) (%)	304 (64.8)	280 (68.3)	281 (67.1)	281 (70.1)	287 (75.1)
Diabetes (%)	37 (4.5)	33 (4.0)	39 (4.7)	39 (4.7)	33 (4.0)
Regular aspirin use (%)	9 (1.1)	19 (2.3)	10 (1.2)	13 (1.6)	14 (1.7)
Family history of intestinal cancer (%)	24 (2.9)	34 (4.1)	29 (3.5)	26 (3.1)	33 (4.0)
Total energy intake*, kcal/day	2098 (819)	2342 (776)	2404 (800)	2508 (759)	2783 (860)
Total FOSs					
Women (%)	547 (65.8)	457 (55.1)	421 (50.7)	348 (41.9)	308 (37.1)
Age, years*	57.5 (10.6)	56.8 (10.9)	56.8 (10.9)	55.8 (11.4)	54.6 (12.3)
Body mass index*, kg/m^2	26.0 (4.1)	26.0 (4.0)	25.8 (4.0)	25.8 (3.9)	25.4 (3.7)
Medium/high physical activity (%)	552 (66.4)	582 (70.1)	562 (67.6)	550 (66.2)	556 (67.0)
Current smokers (%)	241 (29.0)	228 (27.5)	232 (27.9)	240 (28.9)	281 (33.8)
Alcohol drinkers (%)	572 (68.8)	649 (78.3)	656 (79.1)	673 (81.4)	680 (81.8)
Postmenopausal status (in women) (%)	400 (73.1)	323 (70.7)	280 (66.5)	231 (66.4)	199 (64.6)
Diabetes (%)	72 (8.7)	42 (5.1)	37 (4.5)	18 (2.2)	12 (1.4)
Regular aspirin use (%)	7 (0.8)	16 (1.9)	12 (1.4)	14 (1.7)	16 (1.9)
Family history of intestinal cancer (%)	21 (2.5)	30 (3.6)	38 (4.6)	28 (3.4)	29 (3.5)
Total energy intake*, kcal/day	1689 (537)	2136 (530)	2375 (562)	2711 (642)	3223 (912)

* Numbers are means (standard deviations).

Abbreviations: FOS, fructo-oligosaccharide; GOS, galactooligosaccharide.
